# Variational Reconstruction of Left Cardiac Structure from CMR Images

**DOI:** 10.1371/journal.pone.0145570

**Published:** 2015-12-21

**Authors:** Min Wan, Wei Huang, Jun-Mei Zhang, Xiaodan Zhao, Ru San Tan, Xiaofeng Wan, Liang Zhong

**Affiliations:** 1 Nanchang University, Nanchang, Jiangxi Province, P.R.China 330031; 2 National Heart Centre Singapore, 5 Hospital Drive, Singapore 169609, Singapore; 3 Duke-NUS Graduate Medical School Singapore, 8 College Road, Singapore 169857, Singapore; UC Santa Barbara, UNITED STATES

## Abstract

Cardiovascular Disease (CVD), accounting for 17% of overall deaths in the USA, is the leading cause of death over the world. Advances in medical imaging techniques make the quantitative assessment of both the anatomy and function of heart possible. The cardiac modeling is an invariable prerequisite for quantitative analysis. In this study, a novel method is proposed to reconstruct the left cardiac structure from multi-planed cardiac magnetic resonance (CMR) images and contours. Routine CMR examination was performed to acquire both long axis and short axis images. Trained technologists delineated the endocardial contours. Multiple sets of two dimensional contours were projected into the three dimensional patient-based coordinate system and registered to each other. The union of the registered point sets was applied a variational surface reconstruction algorithm based on Delaunay triangulation and graph-cuts. The resulting triangulated surfaces were further post-processed. Quantitative evaluation on our method was performed via computing the overlapping ratio between the reconstructed model and the manually delineated long axis contours, which validates our method. We envisage that this method could be used by radiographers and cardiologists to diagnose and assess cardiac function in patients with diverse heart diseases.

## Introduction

Cardiovascular Disease (CVD), the leading cause of death all over the world, accounts for 17% of overall deaths in the USA. CVD claims more lives than the combination of the next seven leading causes of death. Modern diagnosis and treatment of CVD strongly depend on the aid of medical imaging tools. Numerous advances in medical imaging techniques such as computed tomography (CT) and cardiovascular magnetic resonance imaging (CMR or cardiac MRI) greatly help clinicians to acquire both morphological and functional information regarding the heart.

Visual assessment based on experience used to be clinicians’ primary method to diagnosis medical conditions. High intra-/inter-observer variability of visual assessment made clinicians search quantitative methods with high repeatability and reproducibility. Any such quantitative method has an invariable prerequisite, i.e., an accurate computational cardiac model, the study of which has started since the introduction of the imaging modalities. [1] surveyed the literature of modeling techniques from a variety of imaging modalities.

Most studies of cardiac modeling were focused on the left ventricle (LV). This exclusive preference was due to the physiopathological significance of LV. Early approaches [2] used idealized ellipsoidal shapes and variations [3] to modeling the LV. Those compromise methods were practical in the past, however they are quite oversimplified given nowadays advanced imaging techniques and numerical methods. Similar recent approaches [4–7] used more sophisticated superquadric shapes with more control points to replace the simple ellipsoidal shapes. Images were fitted to determine the shape parameters. The accuracy of results based on the pre-assumption may be limited when handling the highly variable subjects. A few recent approaches [8–11] used image data to fit a population-based (statistical) model, which also suffer from the above limitations.

Besides, most reconstructed models are not complete LV models. The basal short axis imaging plane was usually considered as the top of LV models. Two significant parts in the anatomic definition of LV, i.e., the atrioventricular junction (LV inflow tract) and the aorto-ventricular junction (LV outflow tract), are not included in the reconstructed models. However, modern analysis tools such as finite element methods and hemodynamic simulation requires a complete LV model for computation [12–18]. Two junctions are especially significant for the assessment precision considering the influence from mitral valve and aorta valve to the hemodynamic simulation.

Only a few studies addressed the LV inflow or outflow tract as well as the other chambers and vessels in the last decades. Four-chamber model was reconstructed in [9, 19, 20] and a model containing more components such as coronary arteries was established in [10]. As comprehensive as they are, these models have two limitations. Firstly, some of them are population-based (statistical) model. To adapt them to patient-specific modeling methods is not a trivial task. Secondly, the imaging modalities in both studies were 3D CT or high resolution MR or Diffusion Tensor MR providing a fair image resolution and 3D isotropy. However, CMR is considered as the gold-standard for assessing cardiac anatomy and function due to its ability to capture multi-frames images throughout the cardiac cycle and widely used in current clinical practice.

The major difficulties of the whole left heart modeling based on CMR include the large imaging slice spacing, relating three dimensional anisotropy, and the uncertainty of morphologies of two junction orifices. Researchers in numerical engineering proposed various methods to reconstruct models from contours, which generally use triangulation and meshing technique to determine connections in between neighboring contours [21–25], which however suffer from the inevitable heuristics due to the topology variations or contours sparseness. The demand of a robust and precise reconstruction method motivates this study.

In this study, a novel method is proposed to address the left cardiac modeling problem from a variational approach. Contours were delineated to indicate LV, LA, and AO in both short and long axis images. Contours from different images were registered in the patient-based coordinate system. After a series of preprocessing including contour matching and interpolation, a variational surface reconstruction method was applied. Delaunay based tetrahedral meshes were generated to discretize the underlying space. Graph-cuts were applied to solve the variational problem. The surface model containing the LV, LA, and AO was then extracted from the tetrahedral mesh according to the min-cut. The intersection of the reconstructed surface with the long axis imaging plane was validated against to the manually delineated contours using both curve-based and region-based criteria.

The remainder of this article is organized as follows. Section 2 describes the methodology. Section 3 provides the experimental results and the validation. Section 4 concludes this article.

## Methods

In this study, we tested algorithm on ten healthy volunteers. This study was approved by the Singhealth Centralised Institutional Review Board (CIRB No: 2009/705/C) for human research. All enrolled participants gave written informed consent. The MR data are deposited in hospital and are available for research and education purpose. Cardiac relating measurements for each volunteer/patient were given in Table 1.

**Table 1:** Statistics on Volunteers.

Volunteer	height (m)	weight (kg)	BSA (*m* ^2^)	LV mass (g)	RR (ms)	SBP (mmHg)	DBP (mmHg)
1	1.71	68.2	1.8	102	745	115	63
2	1.8	97.5	2.2	125	1005	123	72
3	1.66	78	1.9	115	780	136	79
4	1.56	59.2	1.6	78	765	111	60
5	1.53	46.5	1.4	66	830	110	67
6	1.47	47.1	1.4	85	880	117	75
7	1.62	66	1.7	120	1085	126	78
8	1.47	55	1.5	67	860	92	55
9	1.6	49.8	1.5	82	1000	100	63
10	1.74	77.7	1.9	105	985	128	77
Average	1.616	64.5	1.69	94.5	893.5	115.8	68.9

### Image Acquisition and Contour Delineation

A 1.5T Siemens scanner with ECG gating was utilized to acquire the cine MR images. Both long axis and short axis images were acquired. A parallel stack of short axis images was acquired from the left atrium to left ventricle apex (8mm inter-slice thickness with no inter-slice gap). Three long axis images were acquired orthogonal to the short axis images, i.e., two chamber view, three chamber view, and four chamber view. The TR/TE/flip angle is typically 68ms/1ms/70°. The field of view was typically 320 mm with spatial resolution of less than 1.5 mm (typically 1.43 mm). Each slice was acquired in a single breath hold, with 22 temporal frames per cardiac cycle.

Both long axis and short-axis MR images were processed in the CMRtools suite (Cardiovascular Solution, UK). Endocardium was delineated by experts for the end-diastole (ED) and end-systole (ES) of each image slice.

**Fig 1:**
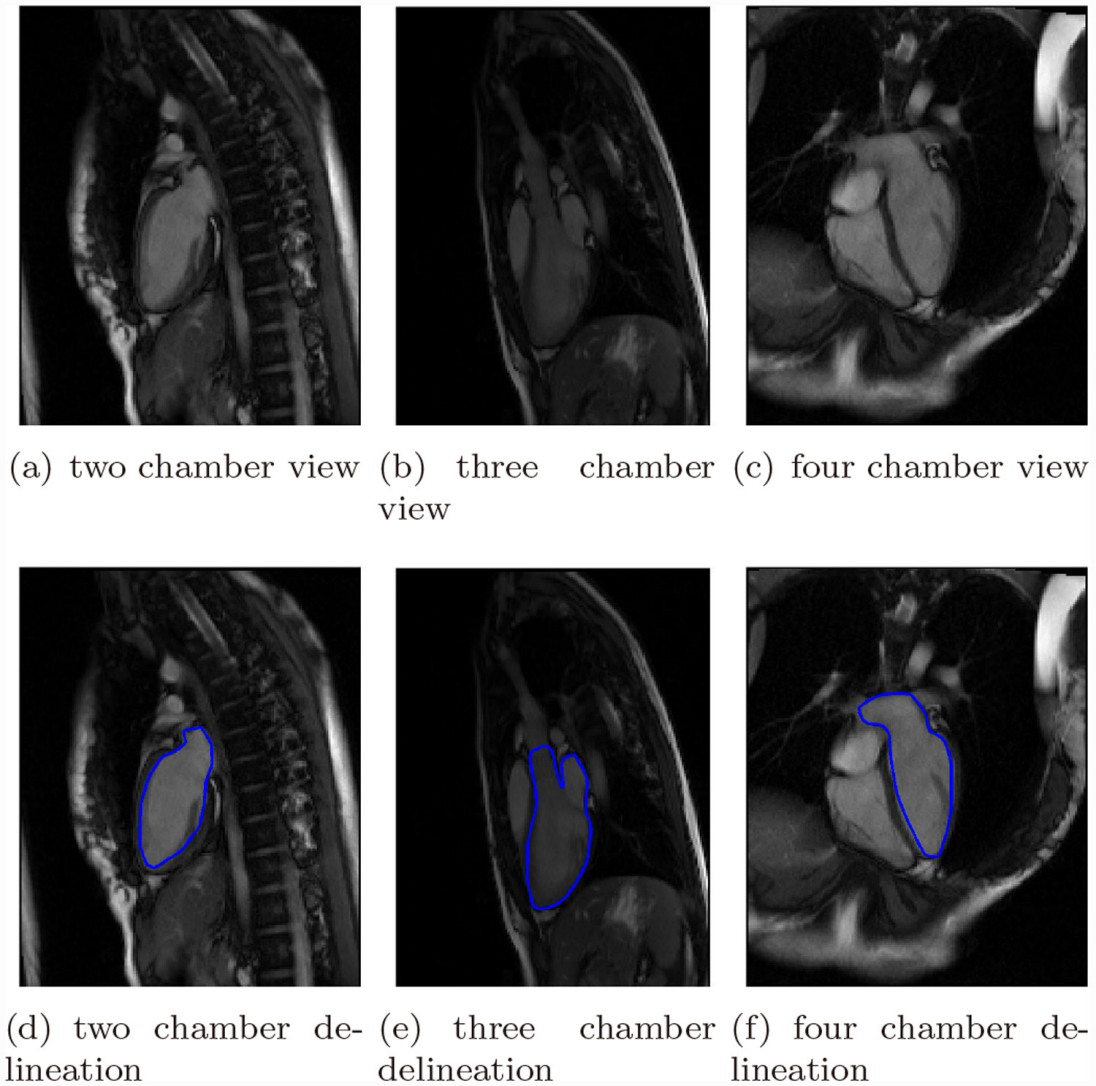
Long axis images and delineated contours.

For the two chamber view, the LV and LA were delineated; for the three chamber view, the LV, LA, and AO were delineated; for the four chamber view, the LV and LA were delineated. Examples of such long axis images and delineations are shown in Fig 1. Identification of the boundaries of LA inflow tract and AO outflow tract could be of less significance. Since the subsequent registration and surface reconstruction procedure would use little information from those.

**Fig 2:**
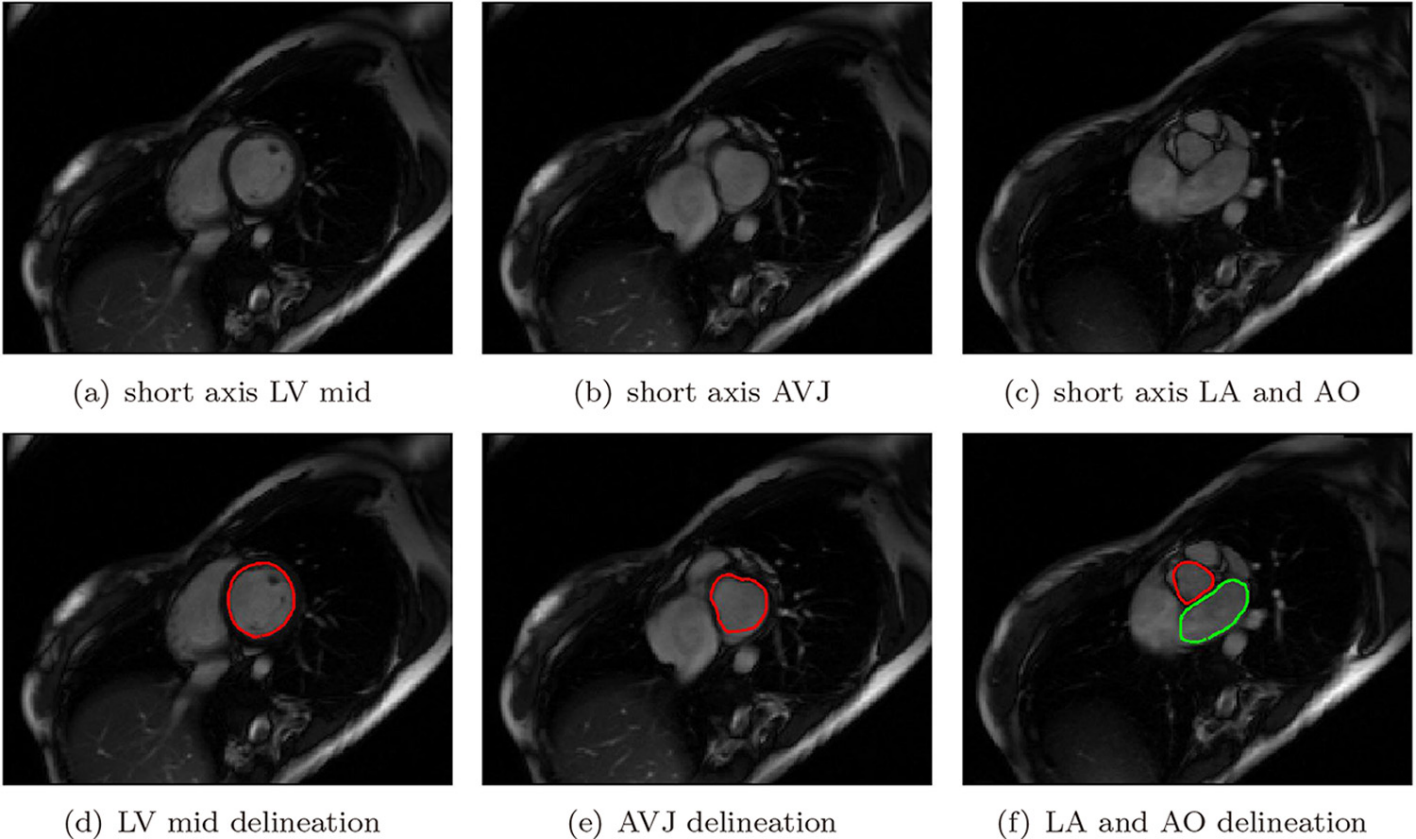
Short axis images and delineated contours.

For short axis images containing only left ventricle, left ventricle endocardium was delineated, see Fig 2(a) and 2(d); for short axis images containing both left atrium and aorta, which could be visibly distinguished, left atrium endocardium and aorta were delineated separately as two disconnected contours, see Fig 2(c) and 2(f); for short axis images containing the LV inflow and outflow tract, i.e., the left atrio-ventricular junction and the aortic-ventricular junction, where the interface between inflow and outflow tracts was not clear, a large contour containing them all was delineated, see Fig 2(b) and 2(e). All papillary muscles were excluded from the myocardial region and were considered as the blood pool instead.

### Point Cloud Registration

The contours delineated from all images were projected into one three dimensional space, i.e., the patient-based coordinate system, according to the imaging specification, e.g., the pixel spacing, the image position, and the image orientation. These image specification are contained in the DICOM file meta information, and the transformation from 2D planar contours to 3D point clouds is as follows.

$$\left[\begin{array}{c} x\\ y\\ z\\ 1 \end{array}\right]=\left[\begin{array}{cccc} U_{x}\Delta u & V_{x}\Delta v & 0 & P_{x}\\ U_{y}\Delta u & V_{y}\Delta v & 0 & P_{y}\\ U_{z}\Delta u & V_{z}\Delta v & 0 & P_{z}\\ 0 & 0 & 0 & 1 \end{array}\right]\;\left[\begin{array}{c} u\\ v\\ 0\\ 1 \end{array}\right]\;,$$ (1)

where

(*u*, *v*) is the 2D coordinate,
(*x*, *y*, *z*) is the transformed 3D coordinate,
(*P*_*x*_, *P*_*y*_, *P*_*z*_) is the image position (cf. DICOM attribute (0020,0032)),
(*U*_*x*, *y*, *z*_, *V*_*x*, *y*, *z*_) is the image orientation (cf. DICOM attribute (0020,0037)),
and (△*u*, △*v*) is the pixel spacing (cf. DICOM attribute (0028,0030)).

**Fig 3:**
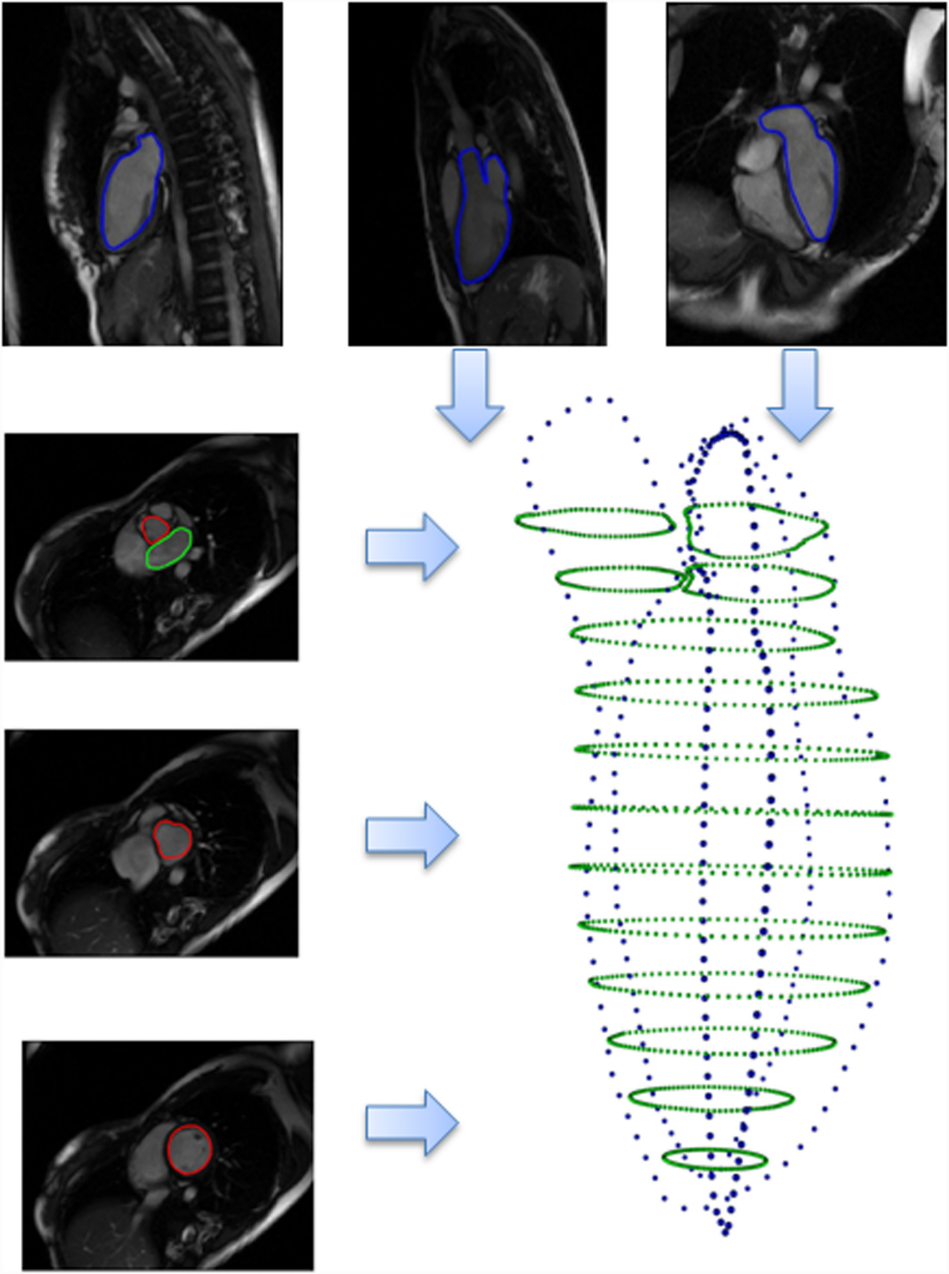
Point cloud from all contours.

Fig 3 illustrates points from contours of all images constituting the point cloud, which approximately profiles the whole left heart structure.

- *C*_*sax*_: the set including all contours on parallel short axis images.
- *C*_2*ch*_: the set from the contour on the two chamber left view image.
- *C*_3*ch*_: the set from the contour on the three chamber view image.
- *C*_4*ch*_: the set from the contour on the four chamber view image.

Simply collecting all contour points for reconstruction is by no means a robust method, considering the breath hold motion and other movements. Certain registration measures must be done before incorporating all contours from different imaging planes.

Recently, the registration methods addressing the misalignment problem could be categorized into two groups. One group of approaches is image-based approaches [26]. The other is geometry-based approaches [27–29]. The image-based approaches are inherently inaccurate due to the large slice spacing and the complex nature of images such as the inhomogeneity and non-uniformity as well as the existence of papillary muscles. Hence, we choose the geometry-based registration method in this stage.

Iterative Closest Point (ICP) algorithm proposed by Besl and McKay [30] and its variations are widely used to register two sets of points, which partially overlap and are usually sampled from one surface.

Directly applying the point cloud registration method to a pair of point clouds may cause artifact registering configuration due to the different data characteristics. Most point cloud registration algortithms were designed for two clouds with relatively dense overlappings, which was not true in our situation. To tackle this issue, we modified the algorithm. Instead of using the whole point clouds, we compute a subset for each point cloud for registration.

The subset filtering criteria are based on the distance between two point clouds.

Let *C*_*X*_ and *C*_*Y*_ be two point clouds, which have limited overlapping part.

$$\begin{array}{l} C_{X}^{{}^{\prime }}=\{x|d\left(x,C_{Y}^{{}^{\prime }}\right)\leq \epsilon ,x\in C_{X}\}\\ C_{Y}^{{}^{\prime }}=\{y|d\left(y,C_{X}^{{}^{\prime }}\right)\leq \epsilon ,y\in C_{Y}\} \end{array}$$

The resulting $C_{X}^{{}^{\prime }}$ and $C_{Y}^{{}^{\prime }}$ have the following properties:

1. $d_{H}\left(C_{X}^{{}^{\prime }},C_{Y}^{{}^{\prime }}\right)\leq \epsilon$, where *d*_*H*_(⋅,⋅) is the Haursdorf distance of two sets.
2. If *ϵ* ≥ *d*_*H*_(*C*_*X*_, *C*_*Y*_), then $C_{X}^{{}^{\prime }}=C_{X},C_{Y}^{{}^{\prime }}=C_{Y}$.
3. If *ϵ*_1_ ≤ *ϵ*_2_, then the resulting $C_{X1}^{{}^{\prime }}\subset C_{X2}^{{}^{\prime }}$.

From Property 2 and 3, the relationship between a proper *ϵ* and the Haursdorf distance between the originial two point clouds is implied. Larger *ϵ* generates larger subsets. Hence, in our experiment, we use *ϵ* = *αd*_*H*_(*C*_*X*_, *C*_*Y*_),0.2 ≤ *α* ≤ 0.5. The empirical *α* is used for the cases when the motion is not significant. The adaptive setting of the *α* parameter taking into account of the initial position of two point clouds is still in study. The algorithm to compute $C_{X}^{{}^{\prime }}$ and $C_{Y}^{{}^{\prime }}$ is described in Table 2.

**Table 2:** Subset computation for two point clouds.

**Inputs**	
1.	Two point clouds *C*_*X*_, *C*_*Y*_
2.	Neighborhood parameter *ϵ*
**Algorithm**	
1.	$C_{X}^{{}^{\prime }}=C_{X}$
2.	$C_{Y}^{{}^{\prime }}=C_{Y}$
3.	While(1)
4.	Find the subset $O_{X}=\left\{x\;|d\left(x,C_{Y}^{{}^{\prime }}\right)>\epsilon \right\}$
5.	Find the subset $O_{Y}=\left\{y\;|d\left(y,C_{X}^{{}^{\prime }}\right)>\epsilon \right\}$
6.	Update $C_{X}^{{}^{\prime }}=C_{X}^{{}^{\prime }}-O_{X}$
7.	Update $C_{Y}^{{}^{\prime }}=C_{Y}^{{}^{\prime }}-O_{Y}$
8.	If |*O*_*X*_|+|*O*_*Y*_| = 0 %%|.| is the cardinality of a set.
9.	Terminate
10.	End If
11.	End While
**Outputs**	Two subsets $C_{X}^{{}^{\prime }}$, $C_{Y}^{{}^{\prime }}$

After computing of such subsets $C_{X}^{{}^{\prime }}$ and $C_{Y}^{{}^{\prime }}$, the classic ICP was applied to them. Assume $C_{X}^{{}^{\prime }}$ is fixed as the reference, the registration configuration could be obtained then: translation **v**, and rotation *R*. The registered subset $C_{Y}^{{}^{\prime }}$ could be represented as $T_{v,R}\left(C_{Y}^{{}^{\prime }}\right)$.

For the four obtained point clouds, i.e., *C*_*sax*_, *C*_2*ch*_, *C*_3*ch*_, and *C*_4*ch*_, the above registration was performed between three pairs among them, namely {*C*_*sax*_, *C*_2*ch*_}, {*C*_*sax*_, *C*_3*ch*_}, and {*C*_*sax*_, *C*_4*ch*_}. *C*_*sax*_ was used for reference in each registration procedure. Note that the selected subset $C_{sax}^{{}^{\prime }}$ for *C*_*sax*_ could be different for different procedure.

**Fig 4:**
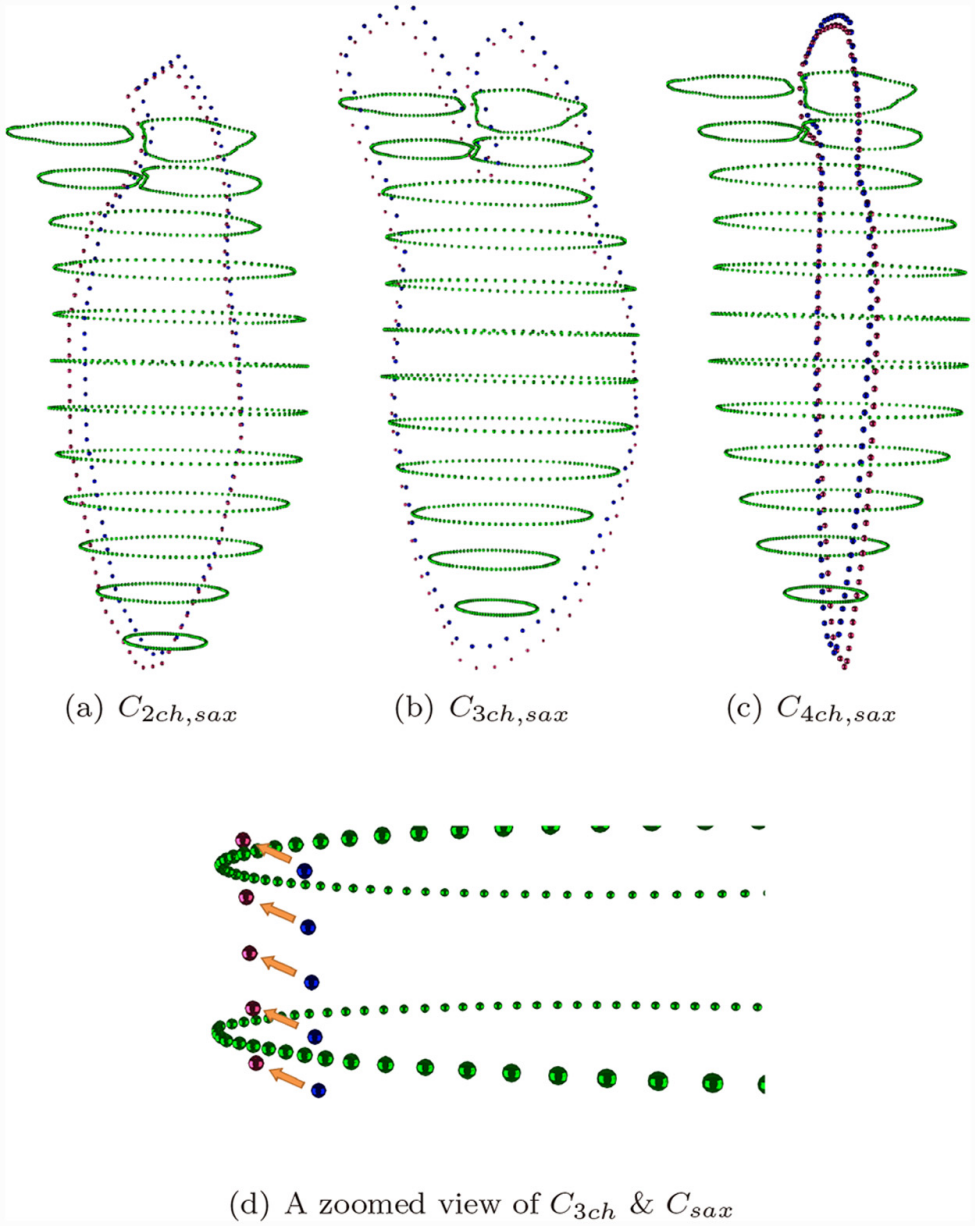
Registration for three pairs, *C*_*sax*_ is in green, original *C*_2*ch*,3*ch*,4*ch*_ are in blue, registered *C*_2*ch*,3*ch*,4*ch*_ are in red.

Three registration configuration were obtained then: (**v**_2_, *R*_2_) for {*C*_*sax*_, *C*_2*ch*_}, (**v**_3_, *R*_3_) for {*C*_*sax*_, *C*_3*ch*_}, and (**v**_4_, *R*_4_) for {*C*_*sax*_, *C*_4*ch*_}. This registration parameters was defined in earlier study. Briefly, *R* is the rotation matrix and **v** is the translate vector. The registration configurations were applied on each long axis point set respectively, obtaining $C_{2ch}^{\prime }$, $C_{3ch}^{\prime }$, and $C_{4ch}^{\prime }$, see Fig 4, where three pairs of registrations are shown respectively as well as a zoomed view.

### Variational Surface Reconstruction

The registered point clouds from long/short axis images were then utilized to reconstruct an endocardial surface of the left heart. The reconstruction task consisted of three steps: (a) interpolation between parallel contour points; (b) tetrahedral mesh generation; (c) variational mesh segmentation and surface extraction.

#### Interpolation between Parallel Contour Points

Short axis contours *C*_*sax*_ were identified as LV, LA, and AO contours *C*_*LV*_, *C*_*LA*_, *C*_*AO*_. The interpolation between parallel contours, i.e., short axis contours, was conducted in each contour group, respectively. The region of the left ventricular inflow tract and outflow tract, i.e., the bifurcation portion, was not interpolated due to the uncertainty of the correspondence between contour points.

The interpolation of contours in each contour group includes (a) contour re-orientation; (b) intra-contour interpolation; (c) contour matching; (d) inter-contour interpolation.

(a) The re-orientation step was adopted to ensure that all contours to be interpolated were counter-clockwise. This could be accomplished by a number of methods. In our approach, we adjusted all contours to be counter-clockwise by making their signed areas to be positive.
(b) The intra-contour interpolation was conducted to make all contours contain the same number of contour points. The interpolation method was chosen as piecewise cubic Hermite interpolating polynomial (PCHIP). Considering the fact that the contours of LV, LA, or AO are nearly circular shapes, smoother interpolation results with $C^{2}$ from a more computationally expensive interpolation method such as cubic spline could not produce a significantly different result compared with PCHIP method.
(c) Contour matching was applied to establish the point-to-point correspondence between two neighboring contours. To obtain a reasonable and most likely correct correspondence between points of two contours, one contour was used as the reference contour and a circular shift was applied on the other one such that the mean of the point-wise distance between two contours was minimized. After the contour matching was applied to all neighboring contours sequentially, all contours in a group (LV, LA, or AO) were reformulated as the format in Table 3.
  In Table 3, *C*_1_, *C*_2_, …, *C*_*L*_ are referred as the *L* parallel contours in a contour group. Each contour contains the same number of contour points, i.e., {*C*_*i*1_, *C*_*i*2_, ⋯, *C*_*iN*_}. Point correspondence was established in each column, i.e., contour points with the same second subscript {*C*_1*j*_, *C*_2*j*_, ⋯, *C*_*Lj*_}.
(d) Using the “vertical” correspondence shown in Table 3, inter-contour interpolation was then conducted in each vertical corresponded point set, i.e., {*C*_1*j*_, *C*_2*j*_, ⋯, *C*_*Lj*_}. The interpolation method was also chosen as PCHIP method. After this inter-contour interpolation, the contour point matrix in Table 3 was enlarged from *L* × *N* to *M* × *N*, where *M* > *N*.

**Table 3:** Re-formulated contour points.

Parallel contours					
*C*_1_	*C*_11_	*C*_12_	*C*_13_	⋯	*C*_1*N*_
*C*_2_	*C*_21_	*C*_22_	*C*_23_	⋯	*C*_2*N*_
⋮	⋮	⋮	⋮	⋮	⋮
⋮	⋮	⋮	⋮	⋮	⋮
*C*_*L*_	*C*_*L*1_	*C*_*L*2_	*C*_*L*3_	⋯	*C*_*LN*_

Note that the intra-/inter-contour interpolation size, i.e., *M* and *N* was determined by the size/density of the tetrahedral mesh generated in the next step. Too large *M* and *N* could produce an almost same result as that of proper *M* and *N*, while introducing extra computation load.

**Fig 5:**
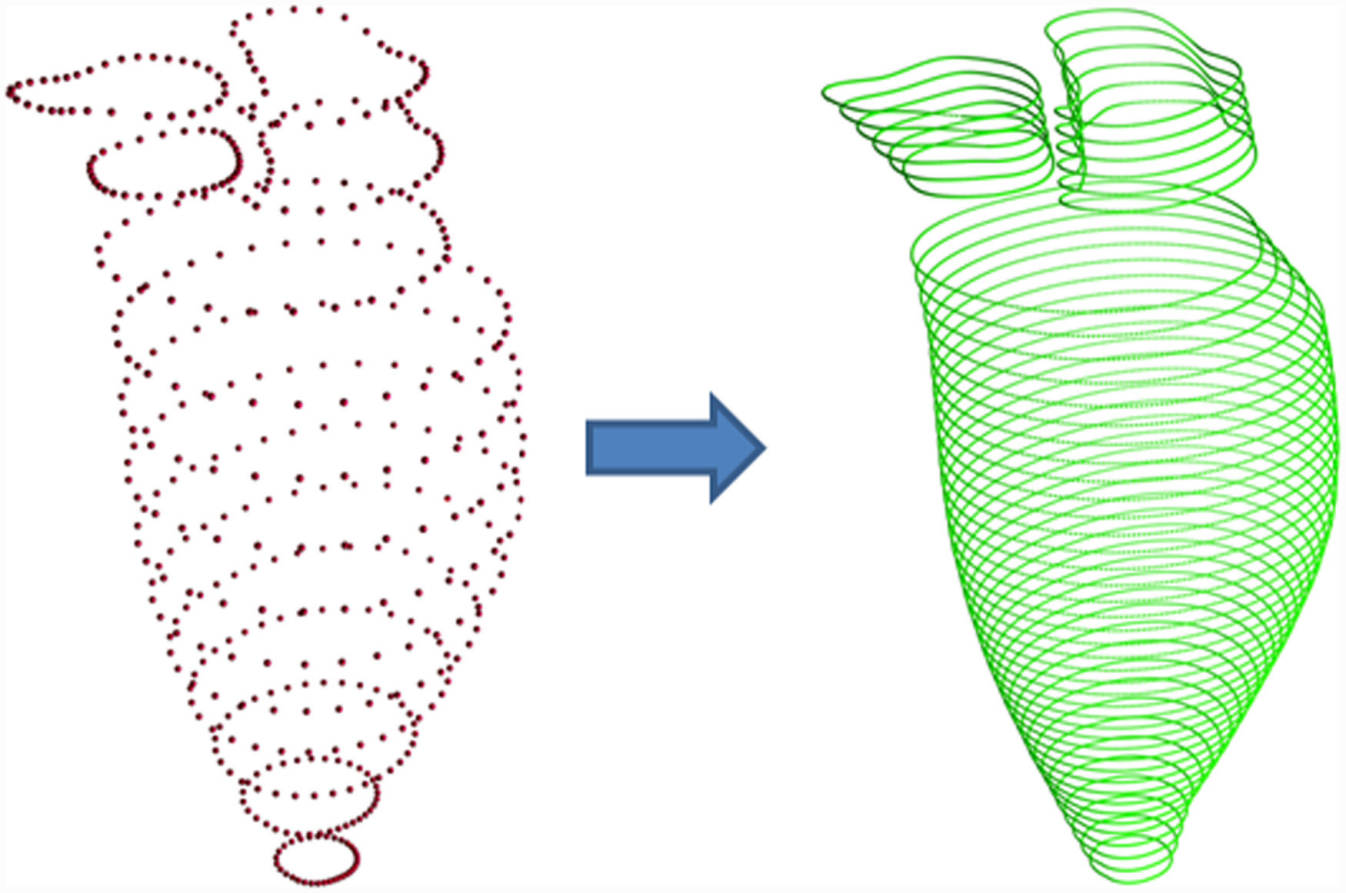
Left: {*C*_*LV*_ ∪ *C*_*LA*_ ∪ *C*_*AO*_}. Right: $\left\{C_{LV}^{\prime }\cup C_{LA}^{\prime }\cup C_{AO}^{\prime }\right\}$. Interpolation was conducted in each short axis group separately. Notice that the region in between groups is not interpolated.

The whole interpolation procedure was conducted on each short axis contour group *C*_*LV*_, *C*_*LA*_, and *C*_*AO*_, obtaining three interpolated and re-formulated point sets $C_{LV}^{\prime }$, $C_{LA}^{\prime }$, and $C_{AO}^{\prime }$. This interpolation step is shown in Fig 5.

#### Tetrahedral Mesh Generation

**Fig 6:**
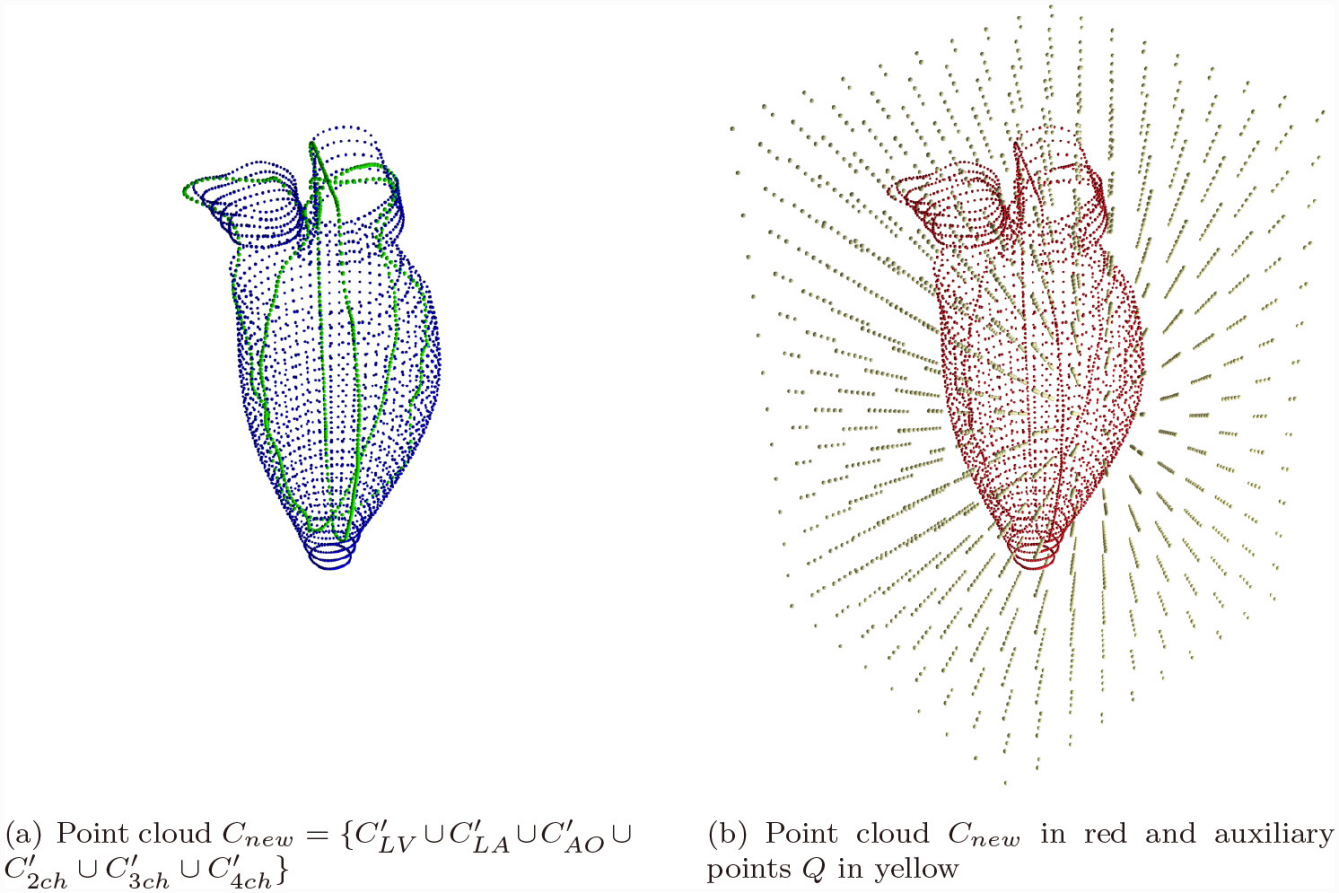
Mesh Generation Preparation. The auxiliary grid points were selected according to the dimensions and the density of the point cloud obtained from interpolation step. *C*_*new*_ and *Q* was used together in the mesh generation.

The registered long axis contour points and interpolated short axis contour points form a new point set profiling the whole left cardiac model with a higher point density: $C_{new}=C_{LV}^{\prime }\cup C_{LA}^{\prime }\cup C_{AO}^{\prime }\cup C_{2ch}^{\prime }\cup C_{3ch}^{\prime }\cup C_{4ch}^{\prime }$, see Fig 6(a).

The point cloud *C*_*new*_ was used to generate a Delaunay-based tetrahedral mesh underlying the region of interest. Auxiliary grid points *Q* was selected according to the dimensions and the density of the point cloud *C*_*new*_, and also inserted during the mesh generation procedure. Fig 6(b) illustrates this preparation for mesh generation, in which the point cloud *C*_*new*_ is annotated in red, while the auxiliary point is in bright yellow. The selection of auxiliary grid points was described in our previous work [31], where the usage of a Delaunay-based mesh was also justified.

#### Variational Mesh Segmentation and Surface Extraction

To reconstruct a triangular mesh surface from the tetrahedral mesh is equivalent to segmenting the tetrahedral mesh into two partitions, interior and exterior. Such task could be addressed as a variational problem, i.e., the weighted minimal surface energy [32].

$$E\left(S\right)=\int_{\Omega }d\left(x,C_{new}\right)dx,$$ (2)

where *d*(*x*, *C*_*new*_) = min_*y* ∈ *C*_*new*__ *d*(*x*, *y*), *d*(*x*, *y*) is the Euclidean distance between *x* and *y*. The surface *S* minimizing this energy functional is the reconstructed surface.

**Fig 7:**
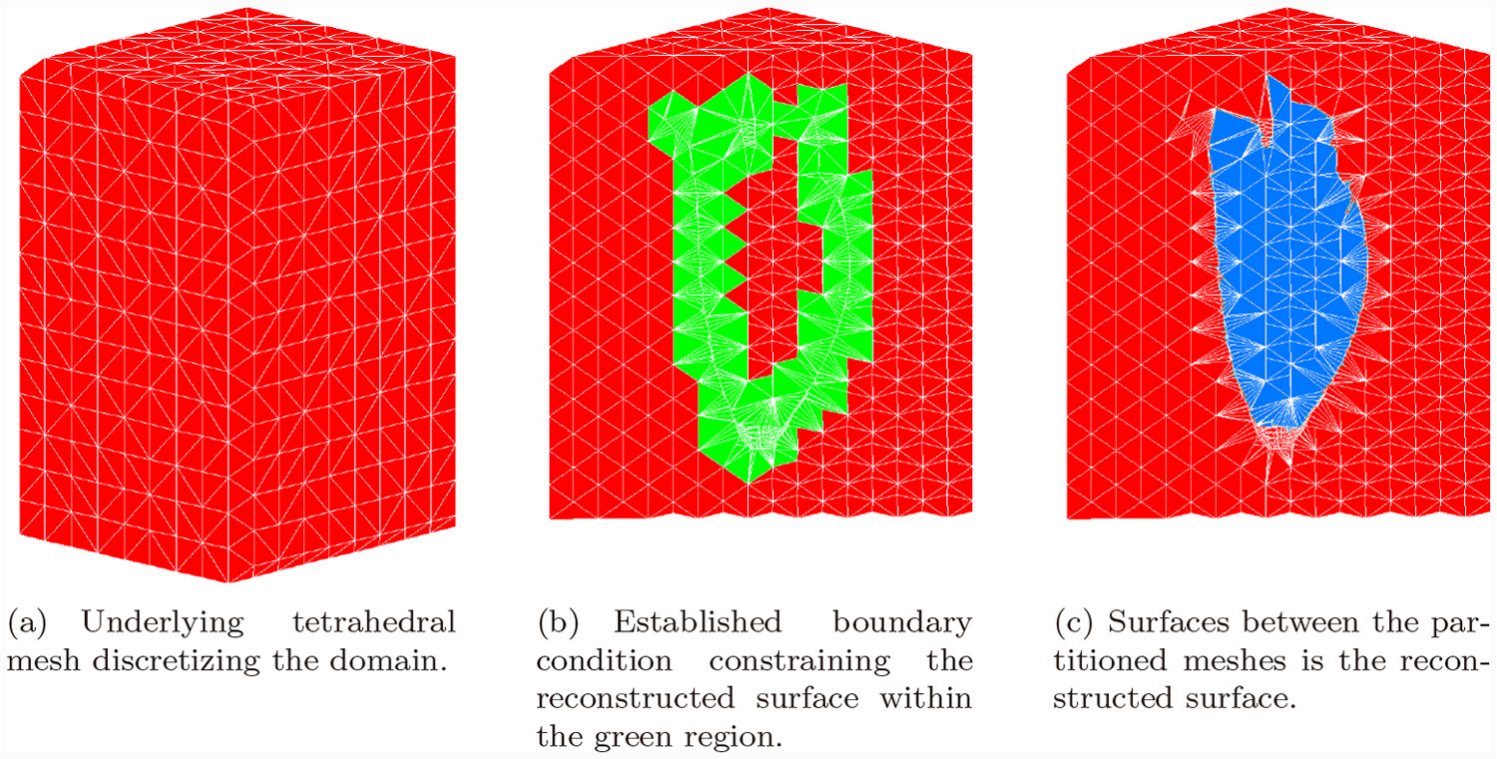
Variational Surface Reconstruction Steps.

**Fig 8:**
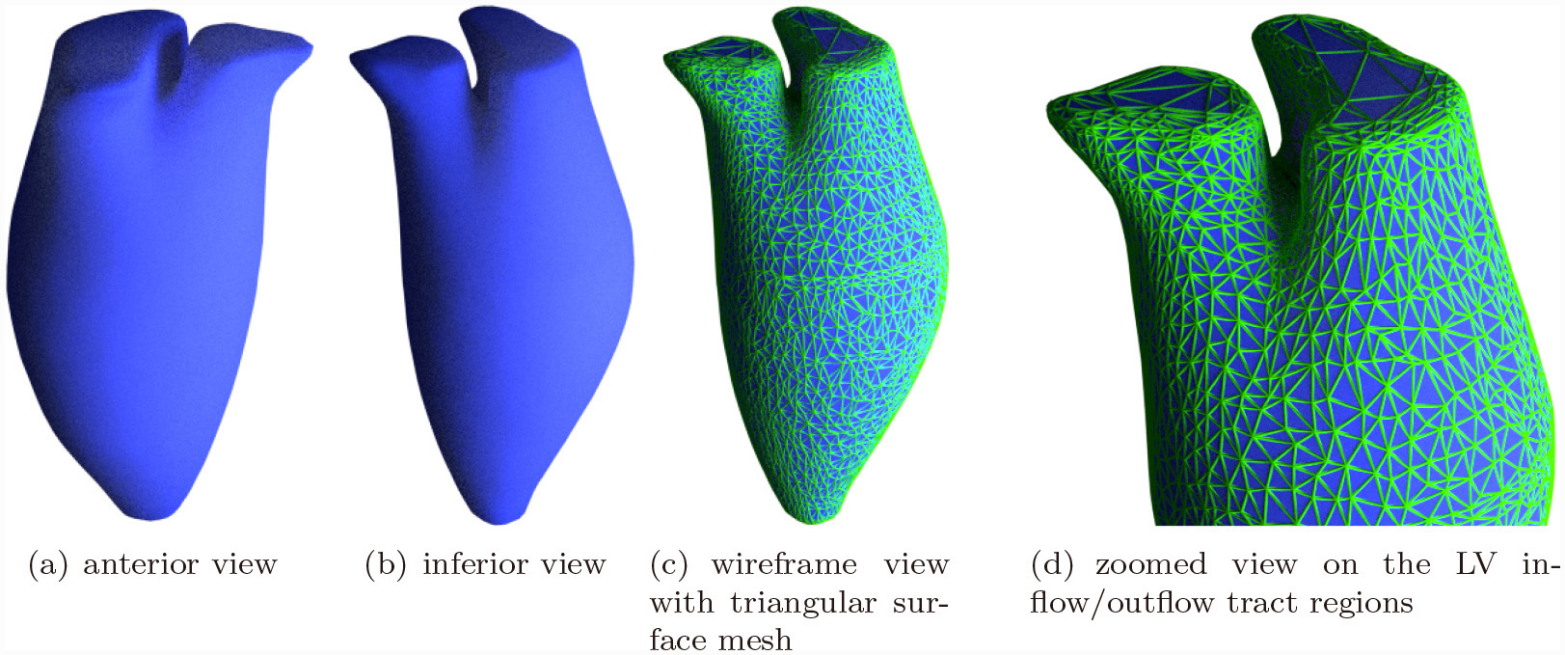
Reconstructed Surface Model of Volunteer 1.

After discretizing this energy functional on the underlying mesh space, it was noted that this minimization problem could be solved by graph-cuts technique [33], i.e., max-flow/min-cut algorithm. In other words, this energy is graph-representable. Solving this problem via graph-cuts has some more technical details to concern, such as determining the solution space and establishing a proper boundary condition for the solver. The operation procedure was also described in our previous study. In this section, we illustrate these steps in Fig 7. Applying the graph-cuts on the problem, a min-cut was then obtained efficiently. A triangular surface mesh was then extracted from the tetrahedral mesh according to the min-cut. After certain post-processing such as smoothing [34] and remeshing [35], a processed left cardiac surface was obtained and an example is shown in Fig 8.

## Results

**Fig 9:**
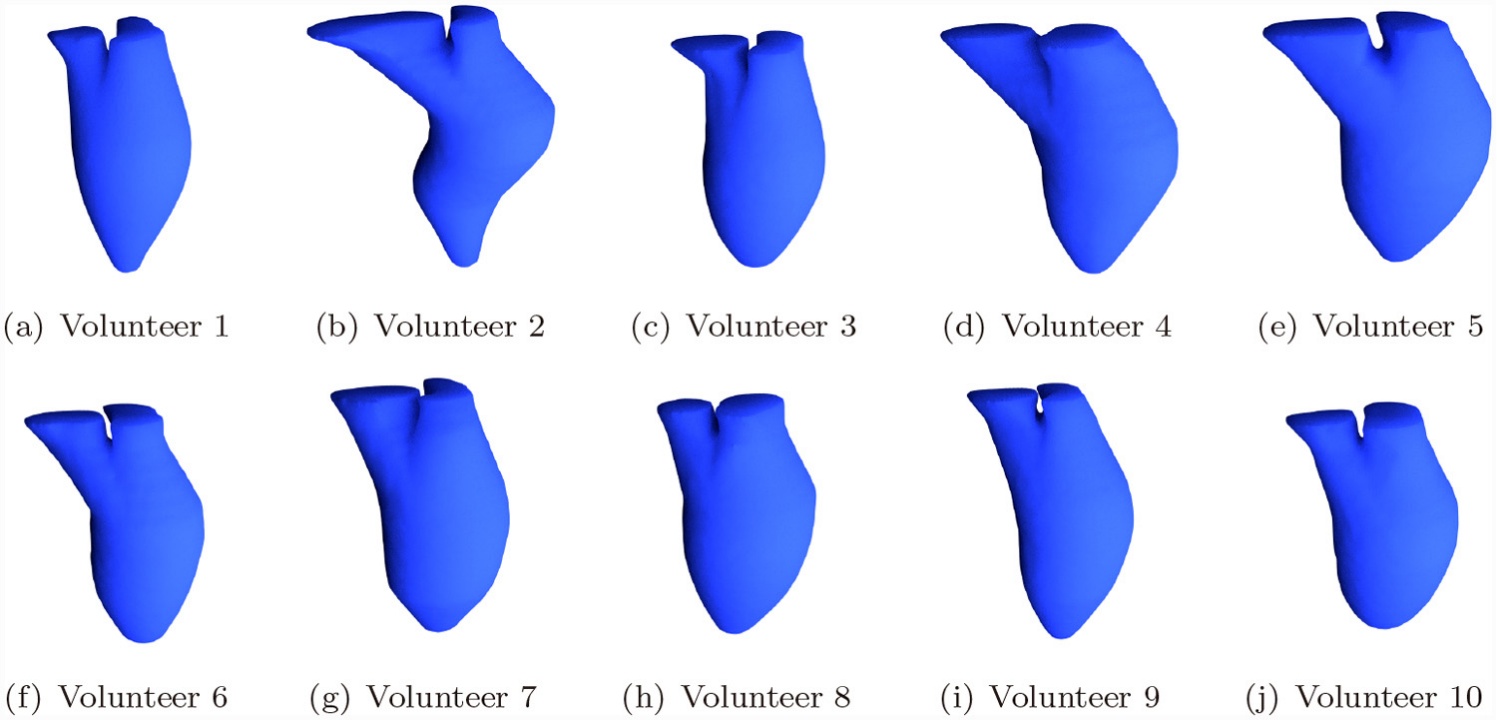
Reconstructed Surface Model of All Volunteers.

The present method was applied on data from ten volunteers. Cardiac relating measurements for each volunteer were give in Table 1. The average time to reconstruct the left cardiac model for one frame is around 6 seconds on a 2.5GHz CPU Desktop. ED and ES frames were reconstructed for each case. The reconstructed surface model of Volunteer 1, ED frame was shown in Fig 8. All ten reconstructed models were shown in Fig 9.

The gold standard to reconstruct the left cardiac model from MRI is not easily accessible. Commercial softwares such as CMRTools usually reconstruct the sole LV model, which is relatively trivial task. Alternatively we use the accurate manually delineated contours to validate the reconstructed model. Since contours were extracted from two-dimensional imaging planes, we projected the reconstructed model to the corresponding planes to produce the sectional contours. Both short and long axis contours should have been used for validation. Since the point cloud contains much more short axis contours than the long axis contours, and interpolation was performed in between short axis contours, the validation result between short axis contours and reconstructed models are extremely good, almost coinciding with each other. Hence, we choose the long axis contours as the validation references, which could evaluate the method more critically.

The overlapping ratio between the reconstructed model and long axis contours was used to evaluate the reliability and accuracy of the reconstruction method. The overlapping ratio is widely used in evaluating the performance of segmentation methods. In our study, the reconstruction task could be thought of as segmenting the left cardiac endocardial cavity in a highly anisotropic 3D image (slice spacing up to 8mm). The long axis contours could serve as the ground truth, against which the reconstruction results could be compared with.

**Fig 10:**
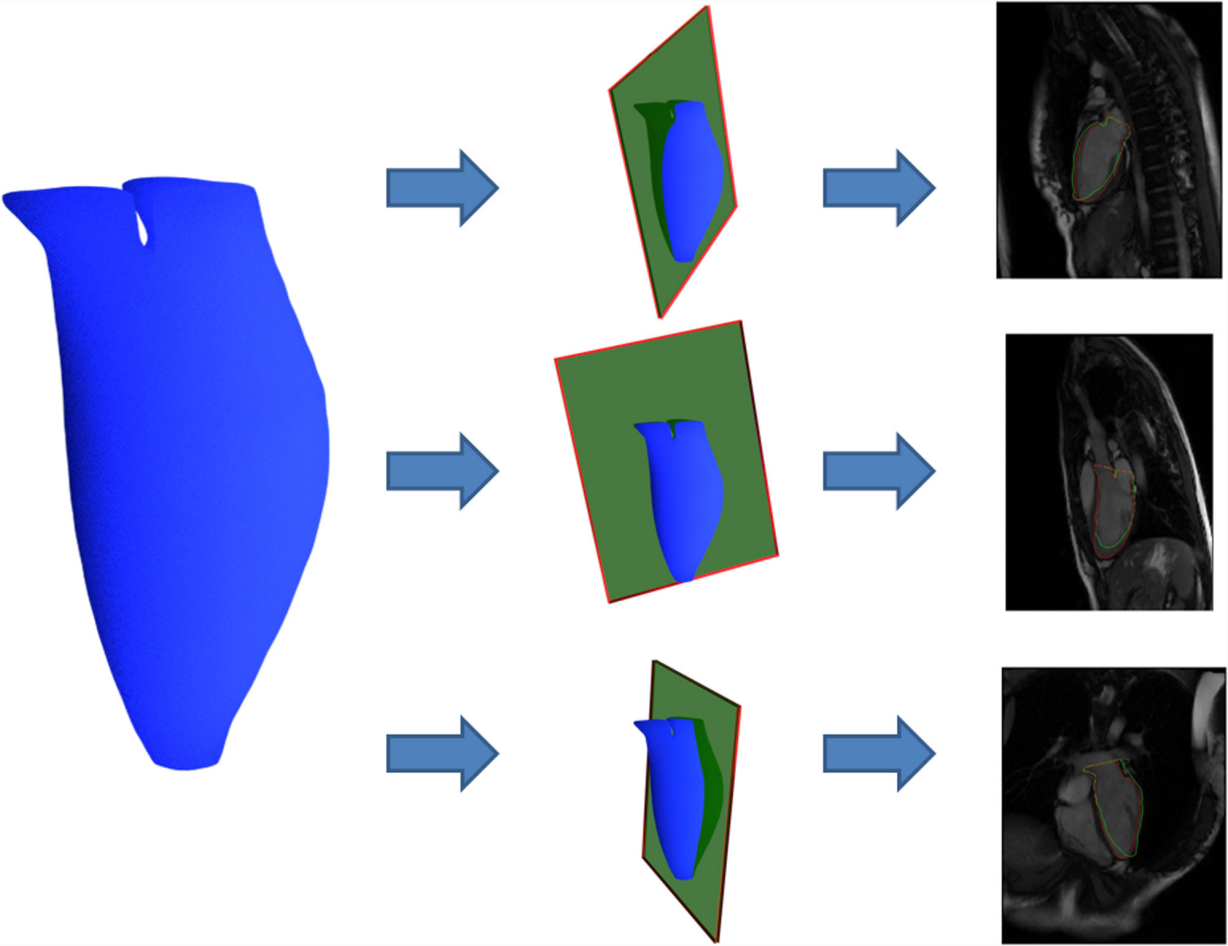
Validation Method: projections of the reconstructed model onto the long axis imaging planes were validated against manually delineated contours.

In the experiments, the intersection of the reconstructed surface model and the long axis imaging planes were computed and validated against the contours drawn by experts in the beginning of the experiments, see Fig 1. This step could be illustrated in Fig 10.

Three criteria were utilized in the evaluation, i.e., Hausdorff distance, Dice similarity Coefficient, Jaccard similarity coefficient. Hausdorff distance is a curve-based coefficient to measure the furthest displacement from the reconstructed model to the ground truth contour.

$$d_{H}\left(X,Y\right)=\inf\left\{\epsilon \geq 0\;;\;X\subseteq Y_{\epsilon }\;\text{and}\;Y\subseteq X_{\epsilon }\right\}$$ (3)

**Fig 11:**
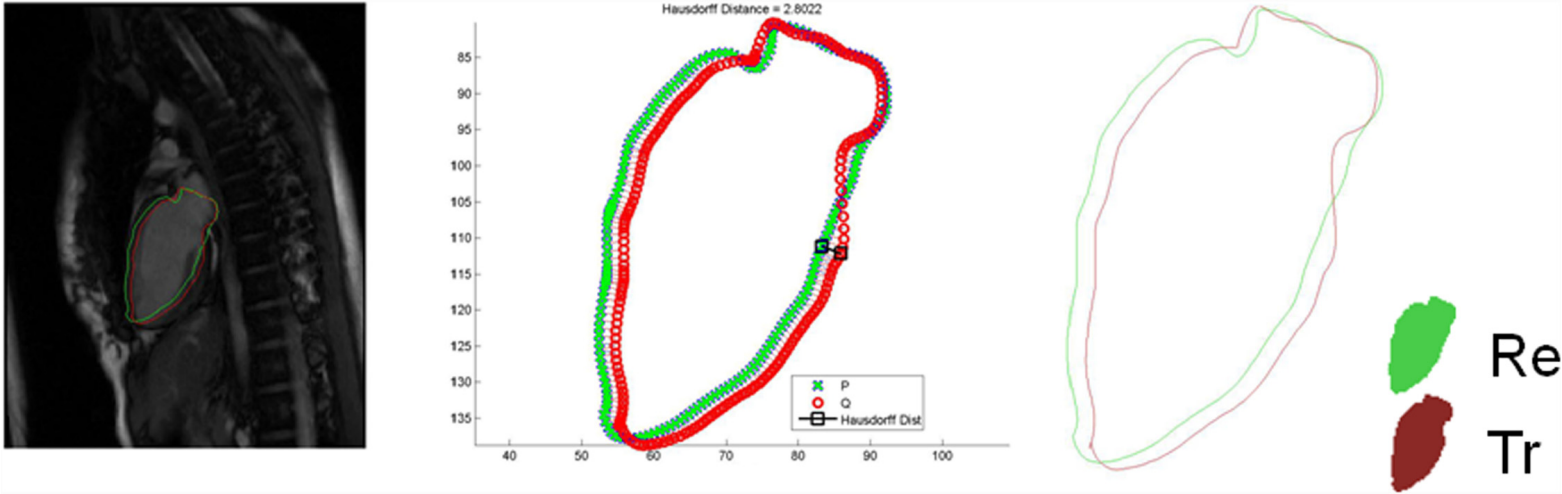
Validation Method. Left: Reconstruction result in green and ground truth contour in red; Middle: Hausdorff Distance between result in green and ground truth in red; Right: Areas bounded by result and ground truth, i.e., *Re* and *Tr* used in Eqs (4) and (5).

Meanwhile, the Dice and Jaccard similarity coefficients are region-based measurements of the overlapping ratio between the reconstructed model and the ground truth contour. The Dice (D) and Jaccard (J) coefficients are defined as follows.

$$D=\frac{2\cdot Area(Re\cap Tr)}{Area(Re)+Area(Tr)}\;$$ (4)

$$J=\frac{Area(Re\cap Tr)}{Area(Re\cup Tr)}\;$$ (5)

where *Re* and *Tr* are areas bounded by the reconstructed model and the manual delineated contour, respectively. A value of 0.7 and above is considered an adequate overlap. An example of the validation against two chamber view long axis contour is shown in Fig 11.

The statistics are given in Table 4 for the validation results of all ten volunteers. The average of all cases are 8.17 for Hausdorff distance, 0.96 for Dice coefficients, and 0.92 for Jaccard coefficients. These results validate our method as a reliable and accurate reconstruction tool.

**Table 4:** The average validation indices for ten volunteers. H: Hausdorff distance; D:Dice similarity coefficients; J: Jaccard similarity coefficients.

Volunteer	two chamber view			three chamber view			four chamber view		
	H(mm)	D	J	H(mm)	D	J	H(mm)	D	J
1	4.00	0.91	0.83	10.57	0.92	0.84	6.62	0.94	0.89
2	9.59	0.93	0.86	6.24	0.93	0.87	11.99	0.93	0.88
3	3.83	0.93	0.87	4.45	0.98	0.97	5.37	0.98	0.96
4	1.49	0.97	0.94	10.84	0.98	0.97	9.02	0.96	0.93
5	6.26	0.97	0.93	8.16	0.99	0.97	5.51	0.95	0.90
6	6.75	0.95	0.91	16.12	0.97	0.95	8.56	0.97	0.93
7	5.11	0.97	0.94	11.71	0.98	0.96	6.13	0.98	0.95
8	13.77	0.92	0.85	9.07	0.96	0.93	10.01	0.95	0.91
9	3.20	0.99	0.97	7.28	0.97	0.94	8.21	0.97	0.95
10	9.47	0.93	0.87	17.32	0.96	0.91	8.44	0.98	0.95

This validation was not performed for the reconstructed model from un-registered point clouds (skipping the step in Section 2.2) to evaluate the impact of the registration on the accuracy. The registration step was designed to correct the motion relating to the breath hold. For the ten healthy volunteers in this study, the motions were relatively subtle. The impact of the registration step is not obvious in this study. In our earlier study, we have encountered data with large distortion from breath hold motions, which critically undermined the accuracy of function assessment. We proposed the method to recover the distorted shape in [29]. The registration step in this article could eliminate this distortion before shape modeling. We will validate this in future works.

Implication: Comprehensive reconstruction of left cardiac structure from CMR images holds several potential implications in heart modelling and function assessment. First, the comprehensive reconstruction of left cardiac structure including left ventricle, left atrium and aorta will straightforward facilitate the downstream heart flow simulation, as reported in our previous publication [12–18]. Second, the reconstruction of left cardiac structure will facilitate rapid automated numerical characterization of point heart surface curvedness and thereby point function spatial-temporal fluctuations of curvedness reflect local heart muscle contraction, allowing comprehensive annotation of regional and global cardiac left ventricular structure and function [36–39] and aorto-ventricular matching before and after heart surgery [40]. Last, this method will contribute a sizeable contemporary CMR imaging atlas of left heart morphology and function in normal subjects and diverse diseased hearts in the near future [41]. These models from CMR images will also be used to validate LV three dimensional echocardiography measurements (i.e., LV volumes and ejection fraction) (3D-echo).

## Conclusions

In this study, we proposed a novel method to semi-automatically reconstruct the whole left cardiac surface model from contours in short and long axis CMR images. Contours from multiple images were registered to each other in the patient coordinate system. Intra/Inter-contour interpolations were performed on the parallel short axis contours then. The resulting contour points were utilized in a variational surface reconstruction via Delaunay triangulation and graph-cuts. The method was validated via evaluating the overlapping ratio between the reconstructed model and multiple long axis contours on the corresponding image planes. High overlapping ratio indicates a reliable reconstruction of the left cardiac structure.

This validation is an alternative method to evaluate our reconstruction method when the gold standard of the left cardiac model was not available in our study. More comprehensive validation will be studied in our future works, including validation against the model reconstructed from CT scanning of the same subjects (which has a higher spatial resolution), and against various medical measurements such as ejection fraction (EF) from CT or cardiac catheterization, or nuclear imaging, the prerequisite of which is an accurate localization and modelling of the mitral valve and aortic valve.
